# Disruptions in cortical circuit connectivity distinguish widespread hyperalgesia from localized pain

**DOI:** 10.3389/fpain.2025.1548500

**Published:** 2025-06-20

**Authors:** George Kenefati, Mika M. Rockholt, Katherine Eisert, Qiaosheng Zhang, Deborah Ok, Christopher G. Gharibo, Lucia Daiana Voiculescu, Lisa V. Doan, Zhe Sage Chen, Jing Wang

**Affiliations:** ^1^ Department of Anesthesiology, Perioperative Care and Pain Medicine, New York University Grossman School of Medicine, New York, NY, United States; ^2^ Interdisciplinary Pain Research Program, New York University Grossman School of Medicine, New York, NY, United States; ^3^ Department of Psychiatry, New York University Grossman School of Medicine, New York, NY, United States; ^4^ Department of Neuroscience & Physiology, Neuroscience Institute, New York University Grossman School of Medicine, New York, NY, United States; ^5^ Department of Biomedical Engineering, New York University Tandon School of Engineering, New York, NY, United States

**Keywords:** chronic pain, chronic low back pain, pain mechanisms, pain phenotyping, hyperalgesia, functional connectivity, EEG

## Abstract

**Introduction:**

This study aims to investigate the interregional functional connectivity in chronic back pain patients with widespread hyperalgesia, patients with localized back pain, and pain-free controls using stimulus-evoked high-density EEG recordings.

**Methods:**

We conducted high-density EEG recordings to compare the functional connectivity and betweenness centrality between these groups.

**Results:**

Compared with controls, chronic pain patients showed altered functional connectivity between regions that process cognitive information and regions that process sensory or affective information. Widespread hyperalgesia, however, is further differentiated from localized pain by decreased inter-hemispheric connectivity of sensory and affective areas and increased intra-hemispheric connectivity between sensory and cognitive cortices. Graph-theoretic analysis showed that whereas chronic pain is associated with decreased centrality of prefrontal, orbitofrontal, and cingulate areas, widespread hyperalgesia is distinguished by increased centrality of prefrontal and insular areas.

**Discussion:**

Together, our results show that although widespread hyperalgesia shares certain features with localized pain, it is further characterized by distinct cortical mechanisms.

## Introduction

Widespread hyperalgesia, heightened pain sensitivity outside the primary area of disease (1–4), is prevalent in pain conditions like osteoarthritis, rheumatoid arthritis, and low back pain (5–8). It correlates with higher pain intensity and daily activity interference, worsening overall health (9). Despite its prevalence and associated morbidity, its mechanisms remain poorly studied.

Imaging studies and serum or plasma markers often don't correlate with widespread hyperalgesia symptoms (10, 11). Given the non-anatomical pain distribution and hypersensitivity, the central nervous system (CNS) is believed to play a key role, with widespread pain or widespread hyperalgesia likely involving nociplastic mechanisms (12–25). Key regions in aversive and cognitive processing of pain include the anterior cingulate cortex (ACC), insular cortex (IC), prefrontal cortex (PFC) and medial orbitofrontal cortex (mOFC) (14, 17, 26–45). The ACC, a hub for aversive processing (26–34, 46, 47), has been shown in animal and human studies to causally regulate generalized aversive response to nociceptive inputs, suggesting its involvement in the mechanisms underlying widespread hyperalgesia (12, 15, 26, 48). The IC is another critical region for aversive processing implicated in fibromyalgia and other rheumatological disorders that show nociplastic features (49–51). Both the ACC and the anterior IC have been shown to process bottom-up aversive experiences (52, 53), while the PFC and mOFC are known to provide top-down cognitive regulation of pain (17, 45, 54–56).

Despite ongoing research, two key questions about widespread pain and hyperalgesia remain unanswered. First, what circuit mechanisms cause disordered nociceptive processing in unrelated, disease-free anatomical regions characterizing widespread hyperalgesia? Second, does widespread hyperalgesia represent a quantitative increase in the strength of maladaptive plasticity in nociceptive circuits already existing in chronic localized pain, or is it caused by distinct circuit changes not seen in localized pain?

Although widespread hyperalgesia may occur in both animals and humans, animal models of widespread hyperalgesia may not accurately reflect clinical conditions. Therefore, human neuroimaging pain studies offer valuable insight and neural biomarkers. While resting-state brain activity provides characterization of functional connectivity (FC), it often lacks specificity due to unknown influences from other cognitive processes. To fully understand the mechanisms of widespread hyperalgesia, it is important to study neural activity specifically during active period of nociceptive processing. Thus, we conducted noxious stimulus-evoked EEG recordings in chronic low back pain patients with widespread hyperalgesia, localized low back pain, and pain-free controls. Our findings indicate that while chronic pain patients exhibit numerous alterations in FC in response to noxious stimuli, widespread hyperalgesia differs from localized pain by distinctly reduced inter-hemispheric connectivity within sensory areas and affective areas. Such inter-hemispheric synchronization deterioration is coupled with an increase in intra-hemispheric connectivity between sensory and non-sensory cortical regions. Furthermore, whereas chronic pain is associated with decreased centrality of the prefrontal, orbitofrontal and cingulate cortical areas, widespread hyperalgesia is characterized by increased centrality of prefrontal and insular cortical areas. Our results indicate that, although widespread hyperalgesia shares certain common features with chronic localized pain at the cortical level, it is also characterized by a distinct set of circuit mechanisms.

## Methods

### Study participants

This study was approved by the New York University Grossman School of Medicine Institutional Review Board (8/22/2019, #i19-01088) and conducted in accordance with the latest version of the Declaration of Helsinki. Written informed consent was obtained from all participants.

Inclusion criteria for chronic back pain patients were diagnosis of chronic low back pain lasting longer than 6 months with a baseline average back pain intensity >4 on a 0–10 numerical rating scale; age between 18 and 75 years; and American Society of Anesthesiologists (ASA) physical status 1–3. Exclusion criteria included acute lumbosacral radiculopathy with sensory or motor symptoms, systemic signs or symptoms, cognitive impairment (by history) or clinical signs of altered mental status; history of schizophrenia; daily benzodiazepine use; and pregnancy.

### Assessment of pain, function, and mood

Prior to EEG recordings, participants underwent a comprehensive assessment of pain, function, and mood based on recommendations from the National Institutes of Health (NIH) Task force on research standards for chronic low back pain (57). PROMIS numeric rating scale—pain intensity, PROMIS pain interference 4a, PROMIS anxiety 8a, PROMIS depression 8a, and PROMIS sleep disturbance assessed symptoms over the preceding week. The McGill Pain Questionnaire short form was used to assess the multidimensional component of pain. PROMIS physical function 4a assessed physical function.

### EEG recordings and mechanical stimulation

Brain activity was recorded using high-density electroencephalography (EEG), equipped with two integrated bipolar leads for vertical electrooculogram (EOG; 64-channel Quik-Cap Neo Net, Compumedics Neuroscan, Charlotte, NC, USA) with the ground electrode positioned on the left cheek. The EEG cap was interfaced with a 64-channel Neuroscan SynAmps 2/RT and Nuevo Amplifier (Compumedics Neuroscan, Charlotte, NC, USA). Each recording session began with two 5-minute baseline recordings (5 minutes with eyes closed, followed by 5 minutes with eyes open) prior to the administration of mechanical stimuli. Participants were blindfolded during the EEG recordings and asked to stay relaxed and in a wakeful state during the behavioral tasks. Weighted mechanical pinprick stimulators (MRC System GmbH, Heidelberg, Germany) exerting forces of 32 mN and 256 mN were used to apply mechanical stimuli both to the lower back and the dorsum of the right hand. 10–20 trials per force were applied at each site with stimulations delivered in random order with an interstimulus interval of approximately 10 s. Participants were asked to rate each stimulus on a 0–10 numeric rating scale, with 0 indicating no pain and 10 indicating high pain. All data were captured using the Curry 8 software (Compumedics Neuroscan, Charlotte, NC, USA) with a sampling rate of 1,000 Hz.

### Pain phenotyping

A threshold for hyperalgesic response to 32 mN stimulation to the hand, a site typically not affected by back pain, was defined as 2 standard deviations above the mean pain rating in control participants. Chronic low back pain participants reporting pain scores below this threshold with 32 mN stimuli to the hand were defined as having chronic localized pain. Chronic low back pain participants reporting pain scores above this threshold with 32 mN stimuli to the hand were defined as having widespread hyperalgesia.

### EEG preprocessing

MNE-Python (version 1.6.1) was utilized for preprocessing (58). First, raw signals were down-sampled to a rate of 400 Hz and a band-pass filter between 1 and 100 Hz was applied. A band-stop filter with 3 Hz width was applied at 60 Hz to eliminate electrical line noise. Noisy EEG channels were identified and subsequently interpolated using PyPREP (59). Criteria for noisy channel detection included low signal-to-noise ratio (SNR), lack of correlation with other channels, low or high relative deviations, presence of high-frequency noise, and poor prediction by other channels based on the random sample consensus approach.

All signals were re-referenced to the average reference. An independent component analysis (ICA) based on the fast ICA algorithm was conducted on the EEG data within the −2.5–2.5 s peri-stimulus time windows. This process utilized a number of independent components (ICs) equivalent to half the number of EEG channels (60). ICs that represented artifacts originating from eye movements, recorded in the EOG electrode, were removed from the EEG data.

The cleaned data were analyzed using functions in MNE-Python, in addition to custom-written Python code. Data were segmented into epochs ranging from −2.5 to 2.5 s in peri-stimulus time. Noisy epochs were identified using the AutoReject package based on Bayesian optimization and were automatically marked for rejection (61). Automatically rejected epochs accurately matched trials marked in the recording notes as containing movement.

To highlight changes in oscillatory activity, epochs were z-scored relative to their pre-stimulus baselines. Z-scored epochs were achieved by subtracting the mean of the baseline period (−2.5–0.0 s) from each epoch (−2.5–2.5 s), followed by division by the standard deviation of the baseline. This procedure ensures a common scale for all epochs, facilitating more accurate comparisons and analyses.

### Source model

To project sensor-space time series to source space, the Minimum Norm Estimate (MNE) was employed, using its implementation in MNE-Python (62). The surface-based, three-shell boundary element model used for anatomical reconstruction was derived from “fsaverage”—a template brain MRI constructed from 40 brain MRI scans (63–65). Loose-orientation was set to 0.2 for inverse solution computation to allow source space dipoles freedom of rotation without deviating extensively from an orientation perpendicular to the cortex. The regions of interest (ROIs) selected for source-localization were the dorsal and rostral anterior cingulate cortices (dACC, rACC), the dorsolateral prefrontal cortex (dlPFC), the medial orbitofrontal cortex (mOFC), the primary somatosensory cortex (S1), and the insular cortex (IC). Both left and right hemisphere regions were considered for connectivity analysis, resulting in a total of 12 regions.

### Source-space frequency-domain representations

To secure a robust stimulus response from each participant, source-space z-scored epochs were averaged. Next, frequency-domain representations were estimated from the averaged source-space z-scored epochs using a batch of multitapers with digital prolate spheroidal sequence (DPSS) windows, one for each of the canonical frequency bands— theta (4–8 Hz), alpha (8–13 Hz), beta (13–30 Hz), low-gamma (30–58.5 Hz) and high-gamma (61.5–100 Hz).

### Functional connectivity (FC) analysis

Connectivity analyses of EEG data were performed using a phase-based approach (66). Phase-based connectivity measures rely on temporal synchronization of brain activity, and thus are more strongly affected by contextual factors. In this study, functional connectivity was investigated using the debiased weighted phase lag index-square estimator (dwPLI) (67). dwPLI is a well-established and highly sensitive phase-based connectivity, a modification of the weighted phase lag index (wPLI), offering a more robust measure against noise and volume conduction effects. It quantifies the asymmetry of phase differences and is debiased to minimize the influence of random phase relationships. The values range from 0 to 1, with 0 indicating either no interaction or symmetric phase differences, and values approaching 1 indicating strong asymmetric phase coupling. Thus, dwPLI is resistant to volume conduction without the risk of reduced sensitivity, as real synchrony at zero phase lag is also discarded. If the wPLI exceeds the phase lag index (PLI), the dwPLI will be negatively biased for small sample sizes, resulting in values below 0.

### Centrality analysis

To better understand the network topology of chronic pain, we analyzed the betweenness centrality of the source space ROIs. Node betweenness centrality, a concept borrowed from graph theory, is the number of shortest paths between pairs of other nodes that pass through a given node. Nodes with high betweenness centrality serve as a bridge between many pairs of other nodes. In our context, each ROI is a node in the pain processing network, and the connections between them, based on dwPLI, are the edges. Regions in the brain with high betweenness centrality act as hubs in the network.

Weighted betweenness centrality was computed using *betweenness_wei.m* from Brain Connectivity Toolbox (15, 68). Because centrality must be estimated from a connection-length matrix, the inverse of each connectivity matrix was taken prior to centrality estimation. The resulting centrality vector (BC, 1xN) is normalized to the range [0,1] as BC[(N-1)x(N-2)], where N is the number of nodes in the network.

### Functional grouping

To reduce the degree of freedom and improve the detection statistics in the presence of multiple comparisons, individual cortical regions were grouped into sensory (S1), affective (ACC and IC) and cognitive areas (mOFC and dlPFC), for both left and right hemispheres. To achieve this, three reduction techniques were tested: mean, median, and maximum. For either FC or centrality measure, the mean, median, and maximum value of the regions within each group were computed, resulting in a connectivity matrix (or centrality vector) with *N* = 6, for the three groups in both hemispheres. Maximum value was selected as the reduction technique for functional grouping.

## Statistical analysis

### Behavioral data

Pain numeric ratings were analyzed using IBM SPSS Statistical Software (Version 28, IBM, New York, United States) and GraphPad Prism (Version 9.4.1, GraphPad Software, Boston, United States). Results were expressed as mean ± standard deviation (SD), standard error of mean (SEM) or median [interquartile range] for continuous variables. Results were expressed as frequency and percentage for categorical variables. An unpaired *t*-test was used to compare mean pain scores of chronic low back pain patients with localized pain vs. widespread pain. *P* < 0.05 was considered significant.

### Analysis of FC and graph-theoretic measures

FC measures were compared using the non-parametric Mann–Whitney *U*-test to account for non-Gaussian distributions in the EEG data of each participant. Results were expressed as mean ± SEM for continuous variables. *P* < 0.05 was considered significant.

## Results

### A subset of chronic pain patients demonstrates widespread hyperalgesia

**Figure 1:**
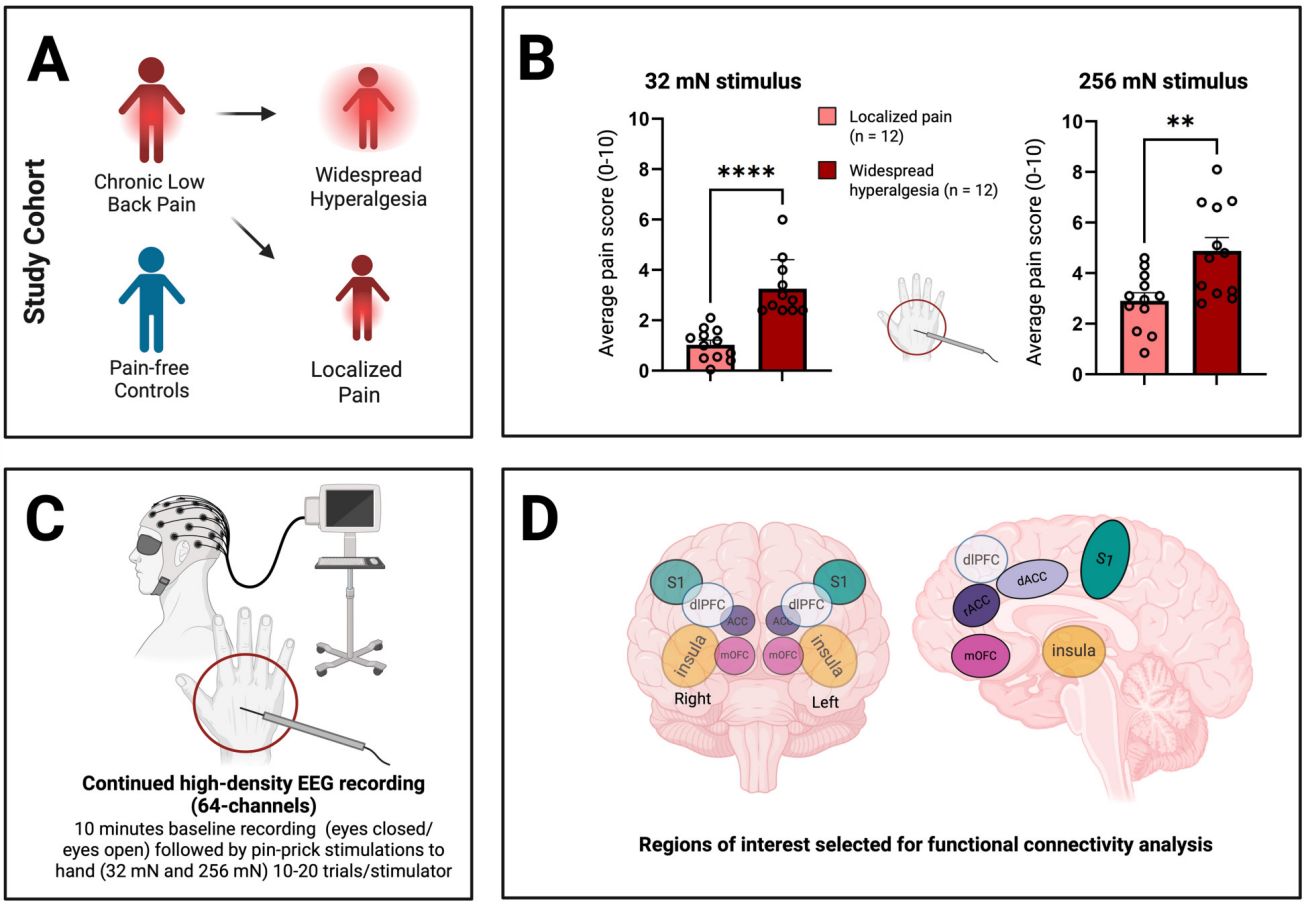
Characterization of patients with widespread hyperalgesia using high-density electroencephalography (EEG) recordings. **(A)** Patients experiencing chronic low back pain, further characterized into those with only localized pain and those with widespread hyperalgesia (see Materials and Methods), as well as pain-free controls underwent EEG recordings. **(B)** Patients with widespread hyperalgesia show increased sensitivity to both 32 mN stimulus and 256 mN stimulus to the dorsum of their hand. Unpaired *t*-test, *p* ≤ 0.05 (*). *p* ≤ 0.01 (**), *p* ≤ 0.001 (***) and *p* ≤ 0.0001 (****). **(C)** Resting-state EEG was collected, including 5-minute intervals with eyes open and 5-minutes with eyes closed. Additionally, EEG recordings were performed during the application of pinprick stimuli (32 mN vs. 256 mN) to the dorsum of the right hand. **(D)** EEG data was analyzed and source localization was performed to isolate cortical areas known to play prominent roles in pain processing (regions of interest, ROIs). These include the primary somatosensory cortex (S1), insula (IC), anterior cingulate cortex (ACC; including rostral ACC or rACC and dorsal ACC or dACC), medial orbitofrontal cortex (mOFC), and dorsolateral prefrontal cortex (dlPFC). Figure creation was assisted by biorender.com.

We used a high-density (64-channel) EEG cap with two integrated bipolar leads for horizontal and vertical electrooculogram (EOG; 64-channel Quik-Cap Neo Net, Compumedics Neuroscan, Charlotte, NC, USA) to measure brain activity before, during and after the application of noxious stimuli to the dorsum of the right hand of all participants (*n* = 43; 24 with chronic low back pain and 19 pain-free control participants; Table 1). None of the participants had chronic pain in the right hand, thus allowing us to evaluate how the presence of chronic pain alters normal cortical nociceptive response or hyperalgesic response. Two calibrated mechanical stimulations were used to provide acute noxious inputs (32 mN and 256 mN). Whereas the 32 mN mechanical stimulation did not trigger pain in control subjects, the 256 mN mechanical stimulation did (17). Next, we separated chronic low back pain patients with widespread hyperalgesia (*n* = 12) from patients with chronic localized low back pain (*n* = 12), using hyperalgesic response to the 32 mN stimulation to the hand as a criterion (Figure 1, Table 1; see Materials and Methods). Patients with widespread hyperalgesia reported higher pain scores for both the 32 mN stimulus and the 256 mN stimulus to the dorsum of their right hand (Figure 1B).

**Table 1:** Demographics and pain characteristics for study subjects.

	Pain-free controls (*n* = 19)	Localized CLBP (*n* = 12)	Widespread pain (*n* = 12)
Demographics			
Gender, *N* (%)			
*Male*	16 (84)	9 (75)	9 (75)
*Female*	3 (16)	3 (25)	3 (25)
Age, mean (± SD), year	47.4 ± 14.5	51.4 ± 16.5	54.3 ± 14.9
Pain characteristics			
Duration of pain, *N* (%)			
*6 months–1 year*	0 (0.0)	0 (0.0)	1 (8.3)
*1–5 years*	0 (0.0)	7 (58)	5 (42)
*> 5 years*	0 (0.0)	5 (42)	6 (50)
Pain frequency/last 6 months, *N* (%)			
*Every* day	0 (0.0)	8 (67)	6 (50)
*At least half the days*	0 (0.0)	3 (25)	5 (42)
*Less than half the days*	0 (0.0)	1 (8.3)	1 (8.3)
*No pain*	19 (100)	0 (0.0)	0 (0.0)
Back pain with radiculopathy, yes, *N* (%)	0 (0.0)	7 (58)	8 (67)
Prior treatments, *N* (%)			
*Opioids*	0 (0.0)	5 (42)	6 (50)
*Psychological counseling*	0 (0.0)	3 (25)	5 (42)
*Exercise therapy*	1 (5.3)	8 (67)	10 (83)
*Injections*	0 (0.0)	6 (50)	7 (58)
Medication quantification scale (MQS)^a^, mean (± SD)			
*NSAIDs* ^b^	0.4 ± 1.1	1.3 ± 2.8	1.7 ± 1.8
*Muscle relaxants* ^c^	0.0 ± 0.0	0.6 ± 1.0	1.4 ± 2.3
*Neuropathic mood agents* ^d^	0.0 ± 0.0	0.9 ± 1.6	0.8 ± 1.3
*Opioids* ^e^	0.0 ± 0.0	2.8 ± 6.7	4.3 ± 5.3
*MQS total*	0.4 ± 1.1	5.6 ± 7.2	8.1 ± 6.9
Pain and quality of life questionnaires			
PROMIS T-score (mean ± SD)^f^			
*8a anxiety*	43.8 ± 7.9	53.9 ± 11.3	58.0 ± 10.6
*4a pain interference*	41.6 ± 0.0	61.4 ± 6.7	63.6 ± 7.6
*4a physical function*	46.1 ± 2.7	38.5 ± 6.7	38.0 ± 7.3
*PROMIS numerical rating scale (0–10)* ^g^	0.1 ± 0.3	5.5 ± 1.5	6.3 ± 2.4
*8b depression*	42.7 ± 6.7	48.0 ± 9.7	54.6 ± 8.0
*Sleep disturbance*	47.2 ± 9.7	52.1 ± 4.0	53.8 ± 10.2
McGill, total score (mean ± SD)^h^	15.0 ± 0.0	30.0 ± 8.8	31.0 ± 11.8
*McGill sensory dimension*	11.0 ± 0.0	23.4 ± 7.0	23.3 ± 9.2
*McGill affective dimension*	4.0 ± 0.0	6.2 ± 2.4	7.7 ± 3.3

^a^ The MQS is a tool to objectively quantify pain by computing numeric values for the patient's pain medication profile, with higher scores representing a higher medication consumption.

^b^ Includes medications such as Ibuprofen, Acetaminophen, Naproxen, Etodolac, Celecoxib, Diclofenac, Aspirin, Indomethacin, Nabumetone, Oxaprozin, Prioxicam, Meloxicam.

^c^ Includes medications such as Baclofen, Carisoprodol, Cyclobenzaprine, Metaxalone, Methocarbamol, Tizanidine.

^d^ Includes medications such as Amitriptyline, Bupropion, Carbamazepine, Citalopram, Desipramine, Doxepin, Duloxetine, Escitalopram, Fluoxetine, Gabapentin, Imipramine, Milnacipran, Nortriptyline, Oxcarbazepine, Paroxetine, Pregabalin, Sertraline, Topiramate, Trazodone.

^e^ Includes medications such as Codeine, Fentanyl, Hydrocodone, Hydromorphone, Meperidine HCl, Methadone, Morphine, Oxycodone, Oxymorphone, Tapentadol, Tramadol.

^f^ With exception of the 4a physical function scale, high PROMIS T-scores mean more of the concept being measured.

^g^ Representing the average pain intensity in the past 7 days, scored from 0 to 10.

^h^ The McGill pain score is used to assess the quality aspects of pain. The higher the total score, the more the pain experience for the patient increases.

### Chronic pain is associated with changes in cortico-cortical connectivity in response to a noxious stimulus

**Figure 2:**
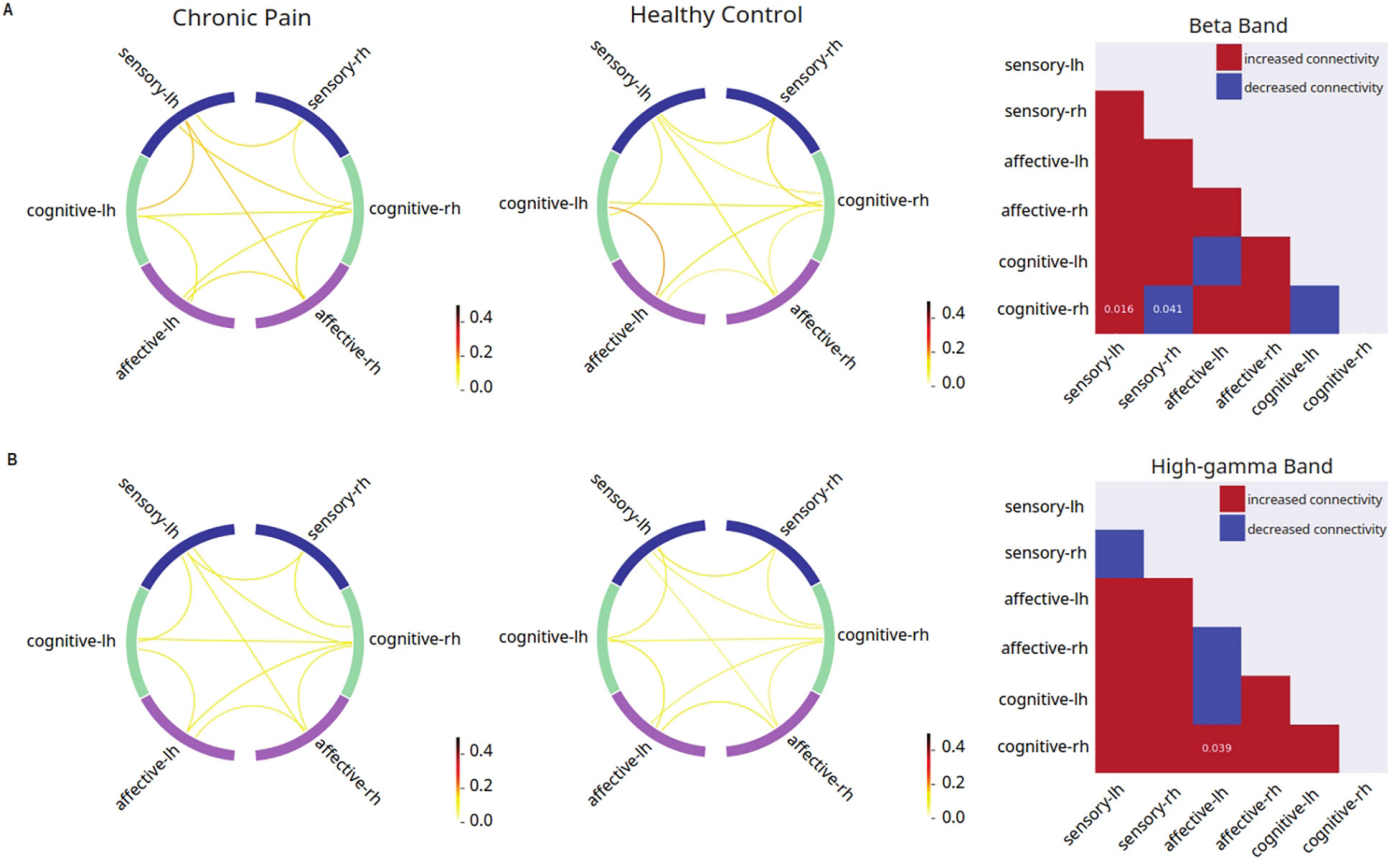
Disrupted mean connectivity across functionally grouped cortical networks are found in chronic pain patients. Left columns: FC between grouped cortical networks in patients with chronic low back pain (*n* = 24); middle columns: FC between grouped cortical networks in pain-free controls (*n* = 19); right columns: matrix comparing mean FC in patients with chronic low back pain vs. pain-free controls (red indicates higher mean connectivity in chronic low back pain patients as compared to controls, blue indicates lower mean connectivity in chronic low back pain patients as compared to controls, *p*-values displayed on matrix indicate statistically significant differences between the two mean connectivities). **(A)** Noxious mechanical stimulation with 256 mN resulted in increased debiased weighted phase lag index (dwPLI) between cognitive-processing cortical areas and the sensory cortex that is contralateral to the stimulus (*p* = 0.016), but decreased FC between cognitive areas and the ipsilateral sensory cortex (*p* = 0.041) in the beta band of the chronic low back pain group as compared to pain-free controls. **(B)** Noxious stimulation resulted in increased dwPLI connectivity between right hemisphere cognitive processing regions and left hemisphere affective processing regions in the high-gamma band (*p* = 0.0388) of the chronic low back pain group as compared to pain-free controls.

We conducted stimulus-evoked EEG recordings in all patients (Figure 1C). After source localization, we examined interregional FC among cortical regions using a phase-coupling method known as debiased weighted phase lag index (dwPLI) (67, 69) (Figure 1D). Pain has sensory, affective and cognitive dimensions, and different cortical regions have primary roles in each of these dimensions. We focused our inquiry on cortical areas known to process these different dimensions of pain: primary somatosensory cortex (S1), ACC, IC, mOFC and dorsolateral PFC (dlPFC) (Figure 1D). Further, to understand how distinct groups of cortices interact at the level of sensory, affective and cognitive dimensions, we adopted a functional grouping strategy in FC analysis in the frequency domain where oscillations at different frequency bands are associated with distinct mechanisms in pain processing. This grouping strategy also enhances the power of our analysis. Thus, we grouped individual cortical regions into sensory (S1), affective (ACC and IC) and cognitive areas (mOFC and dlPFC), then performed FC analysis at the group level. We examined how FC was altered in chronic pain patients in response to a noxious stimulus (256 mN). Here, we found that in response to a noxious stimulus, chronic pain is associated with increased FC between cognitive areas and the sensory cortex that is contralateral to the stimulus, but decreased FC between cognitive areas and the ipsilateral sensory cortex in the beta frequency (13–30 Hz), as well as increased FC between cognitive and affective cortices in the high-gamma frequency (61.5–100 Hz) (Figure 2). In contrast, FC at other frequency bands failed to reach statistical significance.

### A unique pattern of grouped FC distinguishes widespread hyperalgesia from localized pain

**Figure 3:**
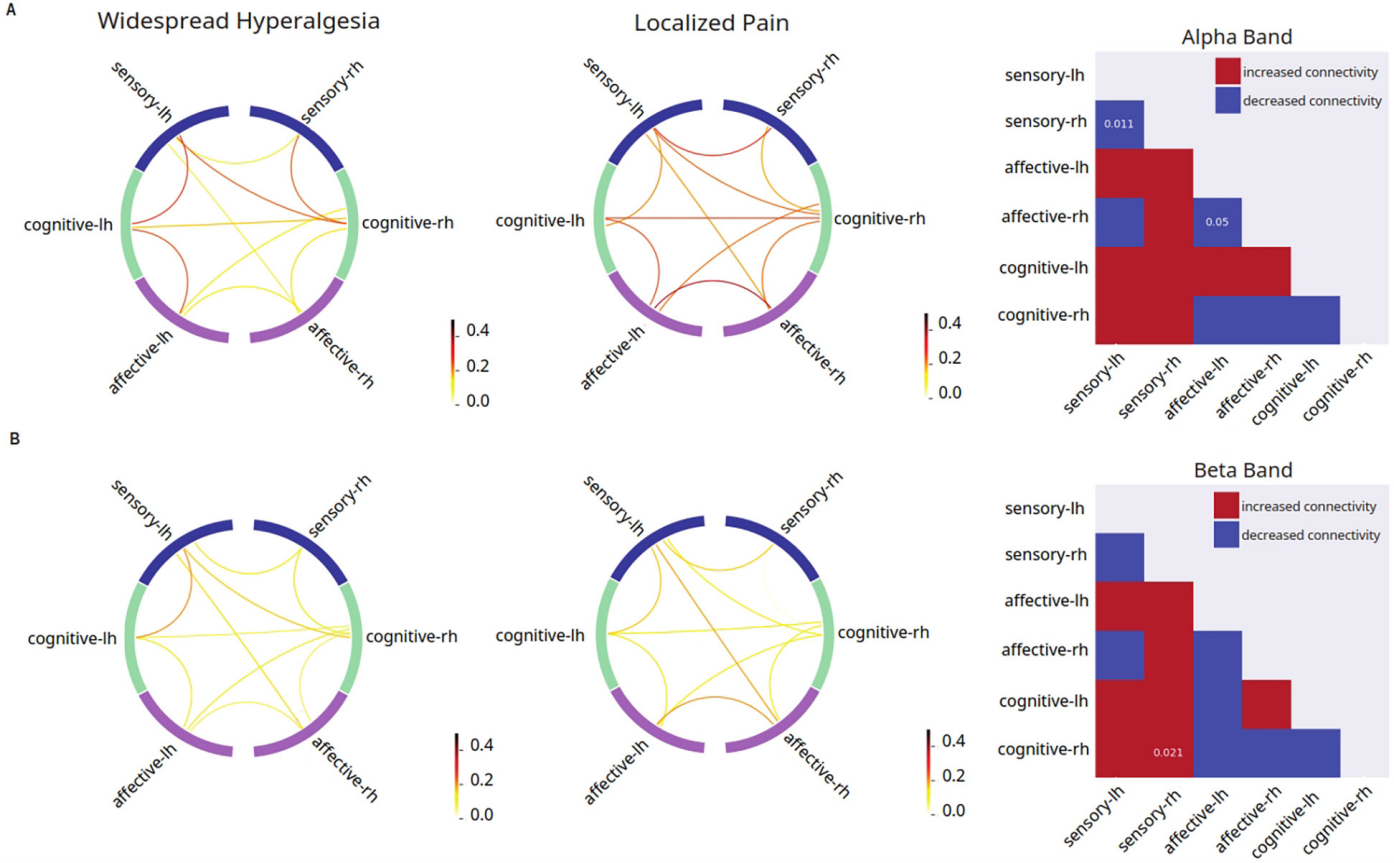
Unique features in mean connectivity across functionally grouped cortical networks in patients with widespread hyperalgesia. Left columns: FC between grouped cortical networks in patients with widespread hyperalgesia (*n* = 12); middle columns: FC between grouped cortical networks in patients with localized pain (*n* = 12); right columns: matrix comparing mean FC in patients with widespread hyperalgesia vs. patients with localized pain (red indicates higher mean connectivity in widespread hyperalgesia patients as compared to localized pain patients, blue indicates lower mean connectivity in widespread hyperalgesia patients as compared to localized pain patients, *p*-values displayed on matrix indicate statistically significant differences between the two mean connectivities). **(A)** Noxious mechanical stimulation with 256 mN resulted in decreased inter-hemispheric dwPLI connectivity between the sensory cortices and affective cortices (*p* = 0.011, *p* = 0.0496) in the alpha band of the widespread hyperalgesia group as compared to the chronic localized pain group. **(B)** Noxious stimulation resulted in increased dwPLI connectivity between the cognitive processing regions and the sensory cortex that is ipsilateral to stimulus in the beta band (*p* = 0.021) of the widespread hyperalgesia group as compared to the chronic localized pain group.

Next, we asked whether widespread pain has its own distinct mechanistic features not found in patients who experience only localized pain (Figure 3). Thus, we conducted grouped FC analysis for widespread hyperalgesia vs. localized pain. We found that, compared with localized pain, widespread hyperalgesia is associated with a number of changes in FC in response to the noxious (256 mN) stimulus. These changes include decreased inter-hemispheric FC between the sensory areas and between affective areas in the alpha frequency (8–13 Hz), and increased FC between the cognitive areas and the sensory cortex ipsilateral to the stimulus in the beta frequency (Figure 3). These results indicate that widespread hyperalgesia has its own mechanistic features compared with localized pain.

### Patients with widespread hyperalgesia display distinct changes in nodal centrality in the cortical functional network

**Figure 4:**
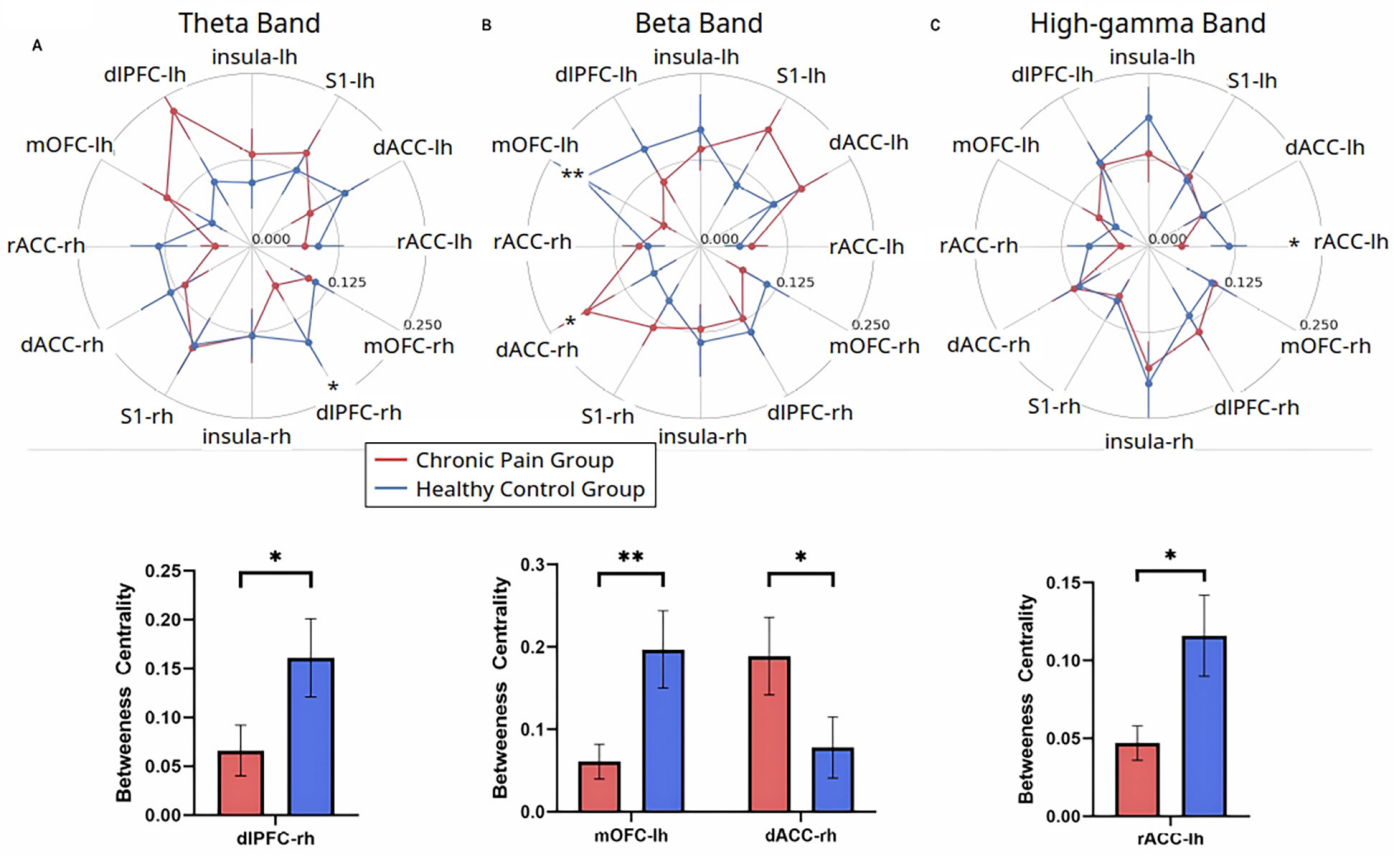
Chronic pain is characterized by changes in node centrality in theta, beta, and high-gamma frequency oscillations. **(A)** Noxious mechanical stimulation with 256 mN resulted in decreased centrality of the right hemisphere dorsolateral PFC in the theta band (*p* = 0.0302), **(B)** Noxious stimulation decreased centrality of the left hemisphere medial OFC (*p* = 0.0095) and increased centrality of the right hemisphere dorsal ACC (*p* = 0.0269) in the beta band. **(C)** Noxious stimulation decreased centrality of the left hemisphere rostral ACC in the high-gamma band (*p* = 0.0441) in the chronic low back pain group (*n* = 24) as compared to pain-free controls (*n* = 19). Data are shown as mean +/− SEM.

**Figure 5:**
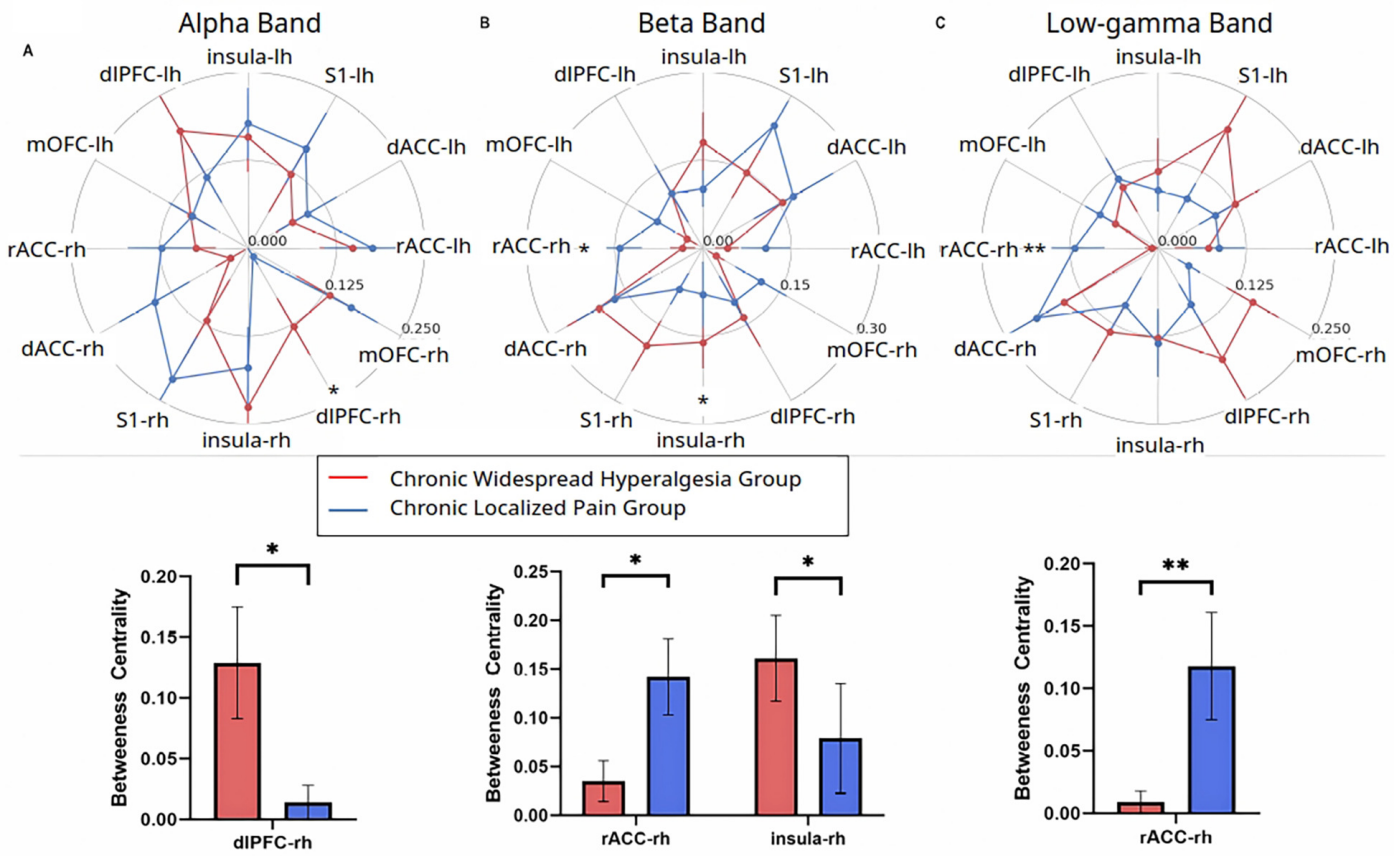
Select features in node centrality further characterize widespread hyperalgesia from localized pain. **(A)** Noxious mechanical stimulation with 256 mN resulted in increased centrality of the right hemisphere dorsolateral PFC in the theta band (*p* = 0.016). **(B)** Noxious stimulation decreased centrality of the right hemisphere rostral ACC (*p* = 0.0275) and increased centrality of the right hemisphere insular cortex (*p* = 0.0483) in the beta band. **(C)** Noxious stimulation decreased centrality of the right hemisphere rostral ACC in the low-gamma band (*p* = 0.0024) in patients with widespread hyperalgesia (*n* = 12) as compared to patients with localized pain (*n* = 12). Data are shown as mean +/− SEM.

To further understand the role of each individual cortical area in driving the overall functional structure in response to noxious inputs, we used an independent graph-theoretic approach to calculate the betweenness centrality, which characterizes how important a cortical area is in organizing a network response to a nociceptive input. We denoted each of the cortical areas (S1, ACC, IC, mOFC, or dlPFC) as a node in pain processing. Our results reveal that chronic pain patients, compared with control subjects, showed decreased centrality of the right dlPFC in the theta frequency (4–8 Hz) (Figure 4A), decreased centrality of the left mOFC but increased centrality of the right dorsal ACC in the beta frequency (Figure 4B), and decreased centrality of the left rostral ACC in the high-gamma frequency (Figure 4C). In contrast, when we compared patients with widespread hyperalgesia with patients with localized back pain, a distinct set of nodal centrality emerged. Here, we found increased centrality in the right dlPFC in the alpha frequency (Figure 5A), increased centrality of the right IC in the beta frequency (Figure 5B), and decreased centrality in the right rostral ACC in both the beta and low-gamma (30–58.5 Hz) frequencies (Figures 5B,C) in the widespread hyperalgesia cohort. These results suggest that different cortical mechanisms may be responsible for widespread hyperalgesia as compared with chronic localized pain.

## Discussion

EEG connectomes offer a powerful tool for studying brain connectivity and advancing our understanding of brain function and dysfunction in both healthy and pathological conditions. In this study, we examined interregional FC among cortical circuits in response to a noxious stimulus in chronic pain patients and pain-free controls. We found that chronic pain patients demonstrated a large number of changes in FC between cognitive-, affective-, and sensory-processing regions in a frequency-dependent manner. More importantly, widespread hyperalgesia, commonly found among chronic pain patients, is further distinguished from chronic localized pain by a specific set of FC changes, indicating unique pathogenetic features.

### Study designs to investigate nociceptive processing in patients with widespread hyperalgesia

Two prominent questions in chronic pain research are (1) what are the circuit mechanisms that give rise to disordered nociceptive processing in unrelated, disease-free areas in the body in chronic pain, and (2) is widespread hyperalgesia a progressively worse condition of chronic localized pain, or is it caused by a distinct set of circuit changes that are not found with localized pain? Several features of our study design enable us to specifically address these two key questions. First, in contrast to prior studies in neuroimaging, which primarily investigate resting-state changes, we focused our inquiry on stimulus-evoked neurophysiological changes. Taking advantage of the temporal precision of EEG recordings, we are able to analyze brain circuits specifically associated with nociceptive processing by performing FC analysis after EEG source localization. Another key feature of our study design is the application of noxious stimuli to a disease-free site, enabling us to investigate generalized, widespread hypersensitivity rather than localized hypersensitivity. Furthermore, comparing patients with chronic localized pain to patients with widespread hyperalgesia, we are able to isolate selective mechanistic features of widespread hyperalgesia.

### Functional connectivity associated with nociceptive processing in chronic pain patients

A key finding in our study is that, in response to a noxious stimulus, there was a large range of changes in FC across multiple cortical areas. This is not surprising, given that nociceptive inputs are processed in a distributive manner throughout the neocortex. The convergence of the nociceptive information carried by distributive circuits is critical for the overall experience of pain, and these circuits may be synchronized by neural oscillations recruited locally that then propagate across long-range projections (70).

Prior fMRI and EEG studies have primarily focused on resting-state FC. In these studies, several featured networks have emerged as highly relevant, including the default mode network (DMN) and salience network (SN) (53, 71). The DMN comprises many of the prefrontal cortical regions and has been implicated in widespread hyperalgesia and pain in fibromyalgia and other rheumatological disorders (49–51). The SN, comprised of the IC and ACC, has also been shown to process the bottom-up sensory stimulus-driven aversive experience, which includes pain (52, 53). Mood disturbances associated with fibromyalgia and other chronic pain conditions have been correlated with changes in the DMN and SN, as well as in their functional connection to the IC and rostral ACC (72, 73).

In contrast to prior studies of resting-state changes in the brain, our study focused on nociceptive processing, specifically in response to a noxious stimulus at a disease-free site, to uncover potential disruptions in brain circuits for endogenous nociceptive processing. Several key regions comprising the DMN and SN, nevertheless, featured prominently in our analysis. For example, we found altered nodal centrality of ACC as well as dlPFC. In addition, we found increased FC between cognitive areas and the sensory cortex that is contralateral to the stimulus, but decreased FC between cognitive areas and the ipsilateral sensory cortex. Our results, in the context of the prior work on resting-state FC, indicate that altered FC at baseline can also translate into disorderly nociceptive processing. These results are further compatible with data from animal models of chronic pain (12, 15, 23).

Cortico-cortical FC can vary from individual to individual, and thus we conducted a functional grouping analysis by combining different cortical areas into groups that process the sensory information (S1), affective information (ACC and IC), or cognitive information (dlPFC and mOFC) of pain. With this analysis framework, we find that chronic pain patients, including those with localized pain and those with widespread hyperalgesia, display a very different FC pattern compared with control subjects. Specifically, chronic pain is associated with increased connectivity between cognitive areas and the sensory cortex that is contralateral to the stimulus, as well as between cognitive and affective cortices. These changes are most prominent in the beta and high-gamma frequencies. Faster frequency (e.g., gamma) oscillations are typically confined to a small neuronal space and are known to involve bottom-up sensory processing, whereas slower frequency (theta, alpha and beta) oscillations are recruited from larger brain networks sometimes associated with persistent brain states such as the state of chronic pain (74–76). Both beta and gamma oscillations in frontal and prefrontal cortical areas have been shown to be positively correlated with ongoing pain (25), whereas gamma oscillations in sensory and prefrontal cortices are known to process sensory and aversive signals, respectively, and have been shown to correlate with evoked stimulus intensity (74, 77–84). Thus, our findings showing alterations in FC in these frequencies indicate enhanced nociceptive processing in response to a noxious stimulus in chronic pain patients.

### Unique mechanistic features distinguish widespread hyperalgesia from chronic localized pain

A key finding in our study is that, in contrast to patients who have chronic localized back pain, patients with widespread hyperalgesia showed additional changes throughout the cortex. At the network level, we found that widespread hyperalgesia is distinguished from localized pain by decreased inter-hemispheric FC between the sensory areas and between affective processing areas in the alpha frequency, and at the same time increased FC between the cognitive processing areas and the sensory area ipsilateral to the stimulus in the beta frequency in response to a noxious stimulus.

Inter-hemispheric FC plays an important role in integrating cognitive, affective and sensory circuits, and is in fact one of the salient and stable features of intrinsic brain function (85). Decreased inter-hemispheric FC has been shown in fMRI studies to have a strong correlation with a number of neuropsychiatric disorders, notably major depression and autism (85–88). There is also emerging evidence from resting-state fMRI studies for disordered inter-hemispheric FC in certain chronic pain conditions (89). Since we did not observe this deficit in inter-hemispheric FC of sensory areas when we compared chronic pain patients with controls, this selective disruption in FC indicates that widespread hyperalgesia is not simply a progression from chronic localized pain but has distinct network features that may be responsible for widespread hypersensitivity.

In addition, our graph-theoretic analysis demonstrated that widespread hyperalgesia is further characterized by increased centrality of the IC. This was not seen among chronic pain patients as a whole. Given the important role of the IC in processing the aversive component of pain (35), this result is not surprising, especially in the context that patients with widespread hyperalgesia are also more likely to experience heightened aversive or affective components of pain and have higher comorbidity with mood disorders (9, 90).

Overall, our results indicate that while some maladaptive cortical circuit disruptions are found in both widespread hyperalgesia and localized pain groups, other cortical mechanisms underlying widespread hyperalgesia may be distinct features not found with localized pain. These results thus shed important light on the mechanisms of widespread or nociplastic pain.

There are several limitations of our study that present opportunities for future studies. First, due to our focus on some of the well-known cortical pain-processing nodes, we did not study FC involving all brain areas critical for pain regulation. For example, subcortical regions which are known to be involved in pain processing include hippocampus, amygdala and nucleus accumbens. Future studies need to examine the roles of these regions in nociceptive functional connectivity, using techniques such as fMRI or intracranial recordings. Secondly, as we grouped distinct cortical regions into networks (sensory, affective and cognitive), there is the possibility that we have reduced the multi-functionality of certain cortical regions. For example, while the IC is known to regulate affective component of pain, studies have shown that it also has a role in sensory processing (91, 92). Likewise, PFC has roles in both cognitive and to a lesser degree affective processing of nociceptive inputs. Thirdly, neuroplasticity is known to play a key role in widespread pain, where both glial cells and neurons can interact to cause persistent synaptic changes in both the brain and spinal cord (6, 93), and future studies are needed to identify the relationship between these cellular mechanisms and the long-range FC identified in the present study. Lastly, more male than female patients enrolled in our study, and thus studies are needed to further characterize sex differences in widespread pain (94).

Our present results indicate that chronic pain causes disruptions in functional connectivity in response to nociceptive inputs. More importantly, whereas chronic pain in general is associated with decreased centrality of prefrontal, orbitofrontal, and cingulate areas, widespread hyperalgesia is further distinguished by increased centrality of prefrontal and insular areas. Therefore, although widespread hyperalgesia shares some features with chronic localized pain, it is also characterized by distinct cortical mechanisms. Insights into these mechanistic differences can enhance our theoretical understanding of chronic pain. At the same time, understanding how disrupted cortical connectivity contributes to widespread hyperalgesia can open new avenues for targeted interventions from a translational point of view.

## Data Availability

The raw data supporting the conclusions of this article will be made available by the authors, without undue reservation.
